# Uniformity Study of Two-Functional Luminescent Dyes Adsorbed over an Anodized Aluminum Coating for Motion-Capturing Pressure- and Temperature-Sensitive Paint Imaging

**DOI:** 10.3390/s18010026

**Published:** 2017-12-23

**Authors:** Masato Ishii, Takeshi Miyazaki, Hirotaka Sakaue

**Affiliations:** 1 Mechanical Section, Second Forensic Science Division, National Research Institute of Police Science, Kashiwa 277-0882, Japan; ishii_masato@nrips.go.jp; 2 Department of Mechanical Engineering and Intelligent System, University of Electro-Communications, Chofu 182-8585, Japan; miyazaki@miyazaki.mce.uec.ac.jp; 3 Department of Aerospace and Mechanical Engineering, University of Notre Dame, Notre Dame, IN 46556, USA

**Keywords:** sensitivity uniformity, PSP/TSP, unsteady measurement, dual luminescence

## Abstract

The pressure- and temperature-sensitive paint (PSP/TSP) technique, for steady-state and unsteady-state measurements, is becoming widespread. However, unsteady quantitative measurement is still difficult because non-uniform distribution of the probes over a test model may cause errors in the results. We focus on the dipping method that applies two luminophores into a binding material to improve sensitivity uniformity over a model surface. A bullet-shaped axisymmetric test model with motion-capturing TSP was used to evaluate the sensitivity uniformity, and three dipping methods (static, convectional, and rotational) were examined. The average peak ratios in the longitudinal direction were 1.17–1.46 for static, 1.38–1.51 for convectional, and 1.42–1.45 for rotational dipping. The standard deviations in the transverse direction were the smallest for rotational (0.022–0.033), relative to static (0.086–0.104), and convectional (0.044–0.065) dipping.

## 1. Introduction

The pressure- and temperature-sensitive paint (PSP/TSP) technique, for the steady-state and unsteady-state measurement of rotor blades, turbomachinery, wing fluttering, and aeroacoustic problems, is becoming widespread [1,2,3,4,5,6,7,8,9,10,11]. One of the unsteady measurement techniques is motion-capturing PSP/TSP, which utilizes luminescent output from two luminophores that have different pressure/temperature sensitivities [12]. In this method, one luminophore provides a signal and the other a reference output. The luminescence ratio distribution between a signal and a reference output on a model surface is converted to a pressure/temperature map by applying calibration data. Yamada et al. [13] and Ishii et al. [14] tried to use the motion-capturing PSP/TSP method to measure the surface pressure/temperature of a bullet-shaped axisymmetric object flying at the speed of sound. However, the pressure/temperature distribution was still aerodynamically incorrect. 

Yamada et al. reported that the luminescence ratio pattern on a projectile surface would cause errors in surface temperature if a single set of calibration data was applied and the pattern had different local sensitivities. Thus, multiple calibration data sets are needed to determine local pressures and temperatures. However, it is difficult to apply multiple calibration data to a moving measurement model because it is not generally possible to fix an accurate position between calibration and experiment without complex image processing. Additionally, the existence of a luminescent pattern would be difficult to distinguish whether the change in luminescent output is based on a non-uniformity of a sensor sensitivity or caused by the physical phenomenon itself. Therefore, a uniformity of a sensor sensitivity over a measurement model needs to be improved. We focused on the dipping method to adsorb luminescent molecules over an axisymmetric test model, since an improvement of uniformity in sensitivity would advance the reliability of the quantitative PSP/TSP measurement.

In previous reports on dual luminescence methods [15,16,17], there were a few discussions about sensitivity uniformity, except for the dual-luminophore PSP/TSP of Mitsuo et al. [18]. They noted the importance of sensitivity uniformity of a model surface but did not evaluate it. Because the motion-capturing PSP/TSP method uses dual luminescence, the evaluation of the luminescence ratio distribution is necessary. Hence, the peak-ratio uniformity for two-color TSP was characterized here for two luminophores applied by a dipping deposition to a binding material. 

## 2. Materials and Methods 

### 2.1. Preparation of Dipping Solutions

Two-color TSP was used to evaluate the sensitivity uniformity over a model surface. Strong luminescent output was obtained with rhodamine B and fluorescein dyes as the signal and reference luminophores, respectively. They were separately dissolved in dichloromethane at concentrations of 0.005 mM (signal) and 0.2 mM (reference). The solutions were then mixed 1:1 by volume.

### 2.2. Preparation of the Test Model

**Figure 1:**
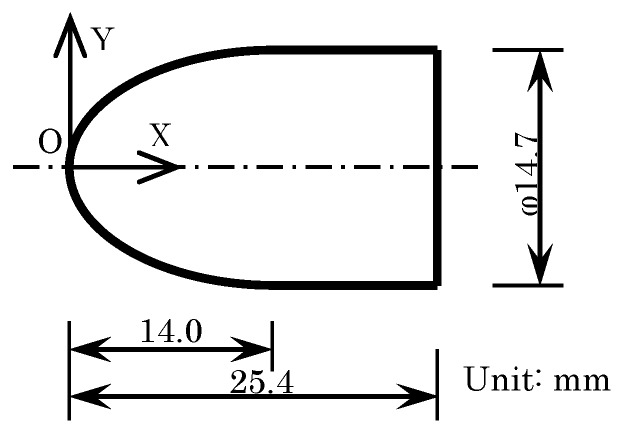
Shape of test model.

Figure 1 shows the shape of the test model as well as the two axes used to compare the results of the dipping methods. The motion-capturing method requires anodization of an aluminum test model. The axes origin was at the top of the model, as shown in Figure 1, where the *X*-axis was along the center and the *Y*-axis was perpendicular to the *X*-axis. 

**Figure 2:**
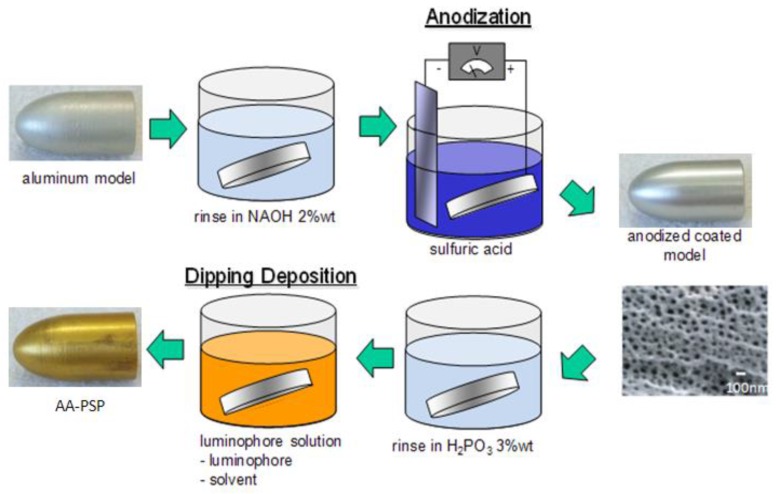
Schematic of the test model preparation.

Figure 2 schematically depicts the anodization, which was generally based on Sakaue’s procedure [19]. At first, the test model was rinsed in a 2 wt % sodium hydroxide solution for 5 m and washed by distilled water. After this procedure was repeated three times, the model was dehydrated by vacuum oven at room temperature. Then, the model was anodized by sulfuric acid with a concentration of 1 mol/L and a temperature of 3 °C for 90 m. The electric voltage was set at a 20 V constant during the anodization. After the anodization, the model was rinsed in a 3 wt % phosphoric acid solution for 20 m with a temperature of 30 °C. Again, the model was washed by distilled water and dehydrated by vacuum oven at room temperature.

### 2.3. Dipping Methods

**Figure 3:**
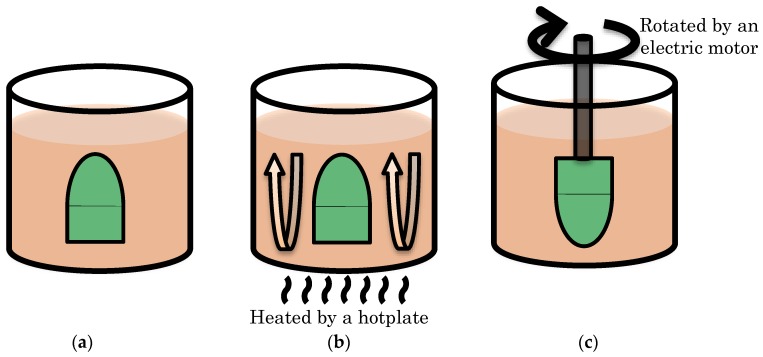
Schematic depictions of dipping methods. (**a**) Static dipping; (**b**) Convectional dipping; (**c**) Rotational dipping.

The sensitivity uniformity was evaluated for three dipping methods depicted in Figure 3: static, convectional, and rotational dipping.

Static dipping, originally used for measurements of a free-flight object, involved dipping with a stationary solution and a stationary test model. The model rested for 5 min at the bottom of a beaker filled with a dipping solution that was not stirred.

Convectional dipping involved heating the solution at the bottom of the beaker during dipping. The solution was heated by a hotplate to a temperature of 35 °C. The stationary test model was then dipped into the solution and rested for 5 min. Here, the solution temperature was kept at 35 °C during dipping. 

**Figure 4:**
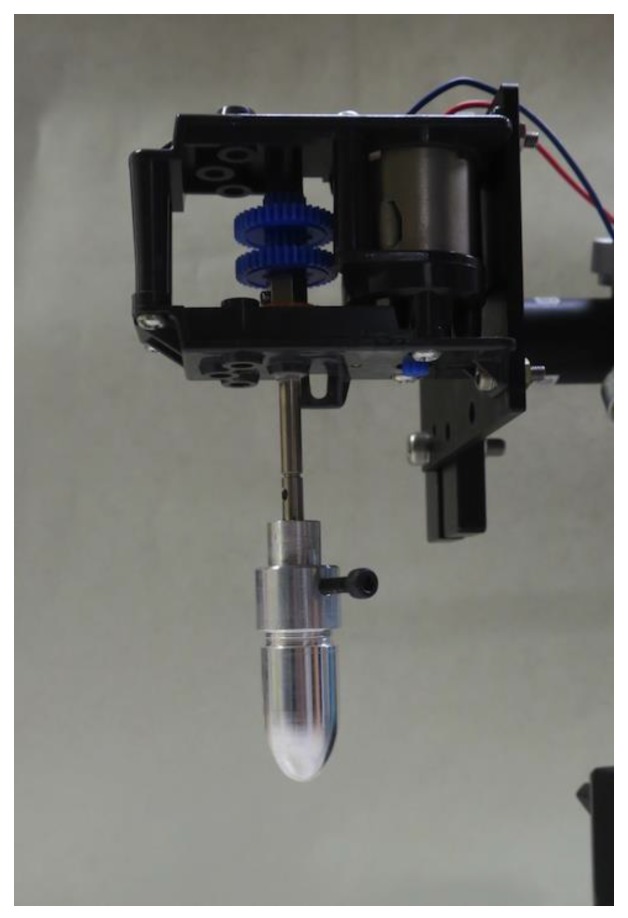
Rotational mechanism for rotational dipping.

Rotational dipping involved rotation of the model by an electric motor and the rotational mechanism shown in Figure 4. The model was held vertically in the solution by a shaft with thread for 3 min at rotated at 870 rpm.

After each dipping procedure, the test model was dried in a vacuum oven at room temperature.

### 2.4. Luminescence Imaging

**Figure 5:**
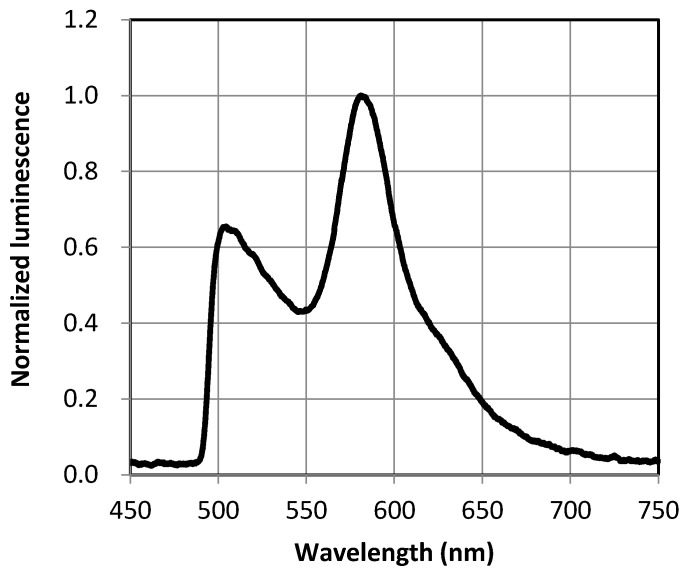
Emission spectra of two-color TSP consists of rhodamine B and fluorescein excited by a 405 nm laser.

The TSP-coated test model was put in the pressure chamber so that the luminescence image of the test model was acquired with a high-speed color camera (Phantom Miro M4, Vision Research Inc., Trenton, NJ, USA). The pressure and temperature of the pressure chamber had set at atmospheric pressure and room temperature. The camera exposure was set at 1.9 ms and the bit depth was 12 bits. The test model was illuminated by a 405 nm laser (BrixX 405-1200, Omicron-Laserage Laserprodukte GmbH, Rodgau, Germany), whose output power was 1.2 W with CW mode, and the laser illumination was expanded to a 50 mm diameter by a collimator lens so that the signal and reference luminophores were excited simultaneously. A 422 nm high pass filter was placed in front of the camera lens to cut off the illumination luminescence. These setups gave a luminescence output from 0 to 4095 pixels. Figure 5 shows that the typical emission spectra of two-color TSP in this study consisted of rhodamine B and fluorescein excited by a 405 nm laser simultaneously. The peak of the spectra around 500 nm was the luminescence emission from fluorescein and the peak around 580 nm was from rhodamine B, respectively.

The luminescence images recorded on the image sensor were divided into red, green, and blue images. The red and green images were then used for measuring the luminescence ratio distributions. The luminescence ratio distribution was taken by simply divided the red image by the green image. All image processing was based on MATLAB 2015b (Mathworks, Inc., Boston, MA, USA).

## 3. Results and Discussion

**Figure 6:**
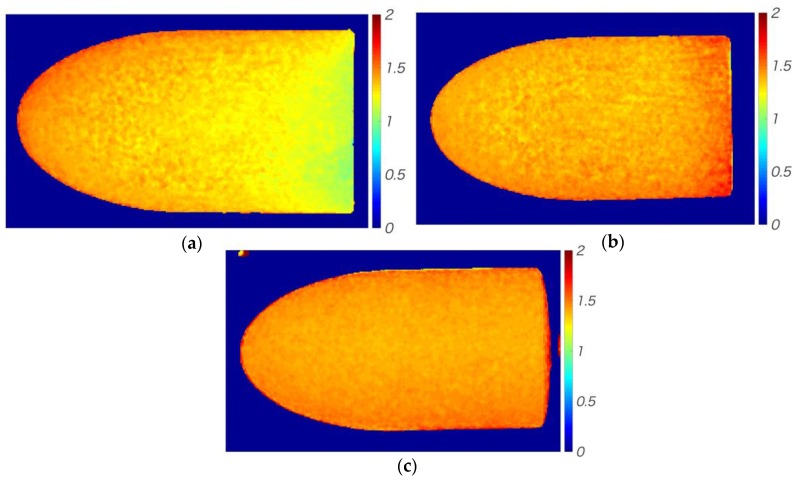
Luminescence ratio distributions on the test models. (**a**) Static dipping; (**b**) Convectional dipping; (**c**) Rotational dipping.

Figure 6 shows the luminescence ratio distributions on the test model surfaces after the different dipping procedures. As shown by Figure 6a, the ratio for the static method was highest at the top of the model and gradually decreased towards the bottom. The ratio obtained by the convectional method (Figure 6b) was highest at the bottom of the model. Finally, the ratio produced by the rotational method was almost constant across the whole surface, as shown in Figure 6c.

**Figure 7:**
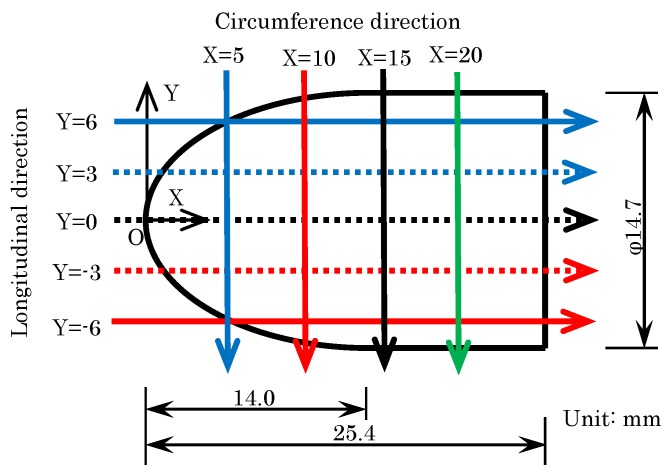
Position of the luminescence ratio profile extraction.

**Figure 8:**
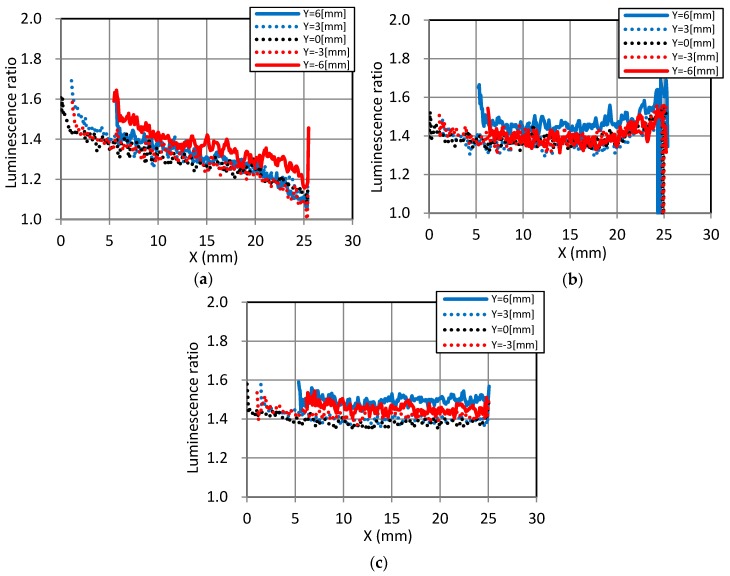
Luminescence ratios along the *X*-axis (longitudinal direction). (**a**) Static dipping; (**b**) Convectional dipping; (**c**) Rotational dipping.

Based on the data in Figure 6, we evaluated the luminescence ratios in detail using the luminescent ratio profiles, and the position of the extraction is shown by the arrows in Figure 7. Figure 8 plots the luminescent ratios along the *X*-axis (longitudinal direction) at Y values of −6, −3, 0, 3, and 6 mm. For static dipping, shown in Figure 8a, the ratios were highest at the top of the model and gradually lower towards the bottom, regardless of the Y-positions. The convectional data in Figure 8b indicates that the ratios were almost uniform along the model surface, except near the bottom where it increased. Finally, the plots of rotational data in Figure 8c indicate almost uniform ratios along the whole model surface. 

**Figure 9:**
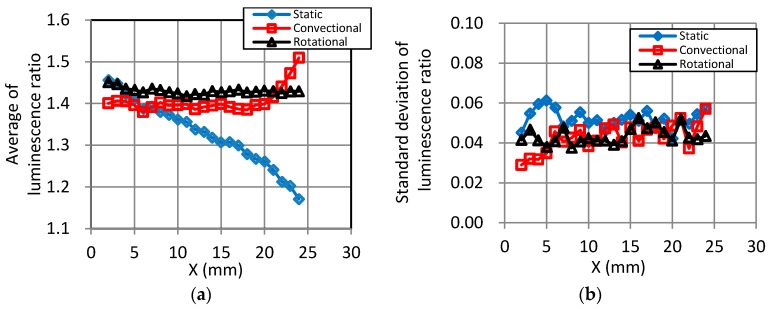
Statistical plots of luminescence ratios along the *X*-axis. (**a**) Average; (**b**) Standard deviation.

Figure 9 shows the statistical plots of the luminescence ratio distributions along the *X*-axis. These lines indicate the averages and the standard deviations of luminescence ratios in the circumference direction over each test models. In Figure 9a, the averaged luminescence ratios for the static dipping method gradually changes from 1.46 at the top of the model to 1.17 at the bottom. In the convectional data (Figure 9b), it changes from 1.38 at the top to 1.51 near the bottom. The rotational data are all within 1.42–1.45, clearly indicating that this dipping method is the most uniform. The standard deviation in the longitudinal direction was similar for all three dipping procedures, as shown in Figure 9b. According to the plot in Figure 9b, there was almost no difference of uniformity in sensitivity between the dipping methods in the circumference direction.

**Figure 10:**
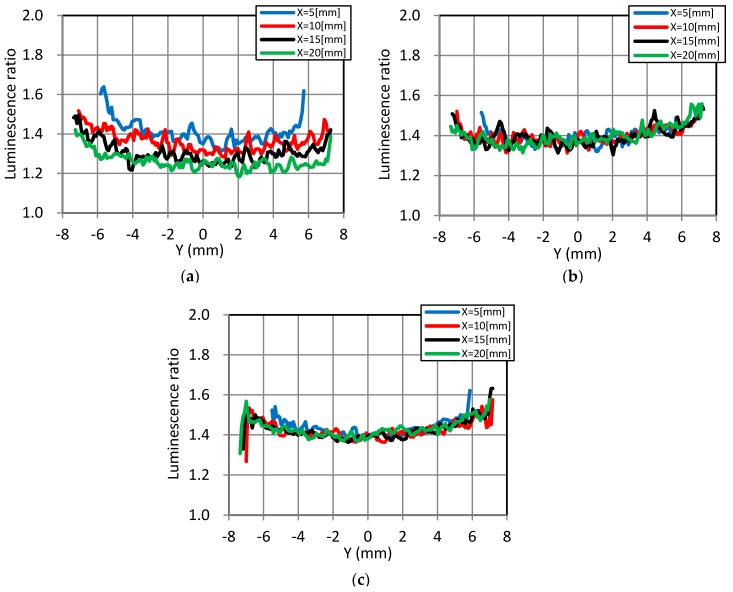
Luminescence ratio plots along the *Y*-axis (circumference direction). (**a**) Static dipping; (**b**) Convectional dipping; (**c**) Rotational dipping.

Figure 10 shows the luminescence ratio plots along the *Y*-axis (circumference direction) at X values of 5, 10, 15, and 20 mm. For the static dipping data, shown in Figure 10a, the ratios decreased with increasing X values. The convectional data in Figure 10b and the rotational data in Figure 10c were almost uniform along with the model surface. 

**Figure 11:**
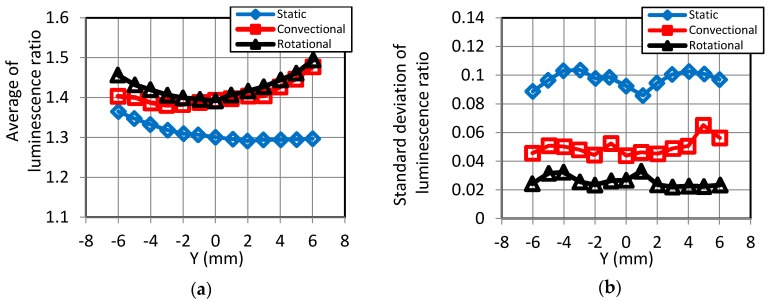
Statistical plots of luminescence ratios along the *Y*-axis. (**a**) Average; (**b**) Standard deviation.

Figure 11 shows the statistical plots of the luminescence ratios along the *Y*-axis. These lines indicate the averages and the standard deviations of luminescence ratios in the longitudinal direction over each test models. Figure 11a indicates that the averaged luminescence ratios obtained with convectional dipping was 1.38–1.48, and those for rotational dipping were similar at 1.39–1.49. In addition, the plots were symmetrical, about Y = 0, because of the configuration of the axisymmetric measurement model. Finally, the averaged ratios for the static method were 1.29–1.37, and these are lower than the others. Figure 11b reveals that the standard deviations for the static, convectional, and rotational dipping methods were 0.086–0.104, 0.044–0.065, and 0.022–0.033, respectively. Hence, the plot in Figure 11b indicates that the standard deviation in the longitudinal direction was lowest for the rotational method and highest for the static method, so the uniformity in sensitivity improved more so by the former method.

Based on these results above, both the convectional and rotational methods, compared to the static method, showed better uniformity in sensitivity. This means that the dipping solution movement affected the luminophore distribution on the axisymmetric test model. Additionally, the rotational method improved uniformity more so than the convectional method. The difference between these two methods is caused by the convection types of the solution. The convection type is classified as natural in the convectional method and as forced in the rotational method. Natural convection generally provides a vertical solution movement, and forced convection provides movement in the vertical and circumference directions. This means that the movement direction of the model in the solution influenced the luminophore distribution. It can be concluded, therefore, that the rotational method produced the most uniform luminescence ratio distribution on the surface of the axisymmetric measurement model. These results showed that adopting the rotational dipping method would solve the non-uniformity of luminophores on an axisymmetric test model such that the effect of error caused by different local sensitivities is eliminated. The improvement of sensitivity uniformity will advance the reliability of quantitative PSP/TSP measurement.

## 4. Conclusions

We evaluated the peak-ratio uniformity of a two-color TSP using an axisymmetric measurement model. Three dipping deposition methods were tested to apply two luminophores onto binding materials. The range of averaged luminescence ratios in the longitudinal direction was 1.17–1.46 for static dipping, 1.38–1.51 for convectional dipping, and 1.42–1.45 for rotational dipping. The averaged ratios in the circumference direction were 1.29–1.37 for static, 1.38–1.48 for convectional, and 1.39–1.49 for rotational dipping. However, the ratio distribution obtained from the static dipping was different from the others. Finally, the range of standard deviation in the transverse direction was smallest for the rotational method (0.022–0.033), compared with those for the static (0.086–0.104) and convectional (0.044–0.065) methods.
